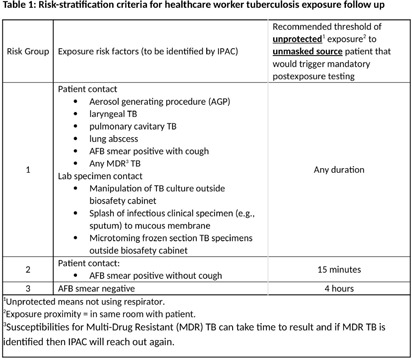# Optimizing tuberculosis exposure investigation amongst healthcare personnel through risk stratification, a tertiary center experience

**DOI:** 10.1017/ash.2025.366

**Published:** 2025-09-24

**Authors:** Ayesha Samreen, Debra Apenhorst, Jenna Rasmusson, Melanie Swift, Aditya Shah

**Affiliations:** 1 Mayo clinic infectious diseases; 2 Mayo Clinic Rochester; 3 Mayo Clinic

## Abstract

**Introduction:** The estimated annual incidence of tuberculosis (TB)in the United States amongst health care personnel (HCP) is low at 2/100,000 persons. Current TB post exposure testing practices may result in many HCP being contacted and tested, with very low yield, thus leading to increased health care resource utilization and HCP anxiety. Based on CDC criteria, Mayo Clinic, Rochester is a medium risk facility. Given that the only transmission we have seen in the last decade is from smear positive, symptomatic patients, we present an alternative, risk-based approach to defining exposure risk to guide followup testing for health care personnel exposed to TB patients. Our goal was to account for the most common exposure follow up (EFU) scenarios and not the rarest situations, which would require case by case discussion. We present a novel risk stratification definition for EFU testing at Mayo Clinic, Rochester and present 12 months’ worth data pre and post initiative. **Methods:** Prior to July 2023, case exposure definition for screening was broad without clarity on duration of exposure or risk for acquisition of the disease. After the new definition was proposed in collaboration with Infection prevention and control (IPAC), Occupational safety and health, and Minnesota department of health, each case was reviewed to determine appropriateness of HCP exposure testing **Results:** In the time frame from July 2022 through June 2023, total of 5 EFUs were conducted, and 70 healthcare personnel were exposed (14 per EFU), and none developed TB infection [MS1] After implementation of new protocol, during July 2023 through June 2024, there were 11 EFUs, 102 healthcare personnel were identified as exposed (9 per EFU), and none developed TB. Of note, the low number of exposure investigations prior to July 2023 coincides with the universal [MS2] masking policy related to the COVID-19 pandemic. **Conclusion:** Existing public heath guidelines do not establish minimum exposure time warranting follow up testing for tuberculosis amongst HCP. However, not all cases need extensive case management as this may lead to excessive costs and resources for testing, conducting EFUs and anxiety amongst HCP. With our proposed exposure risk stratification, we aim to not only reduce resources and time needed to conduct EFUs, but also decrease incorrectly identified HCP to assure the correct ones are being tested. We will continue to audit and review our data at regular intervals with continued feedback and discussion with stakeholders to adopt a more data driven approach to TB exposure followup.

**Figure:**
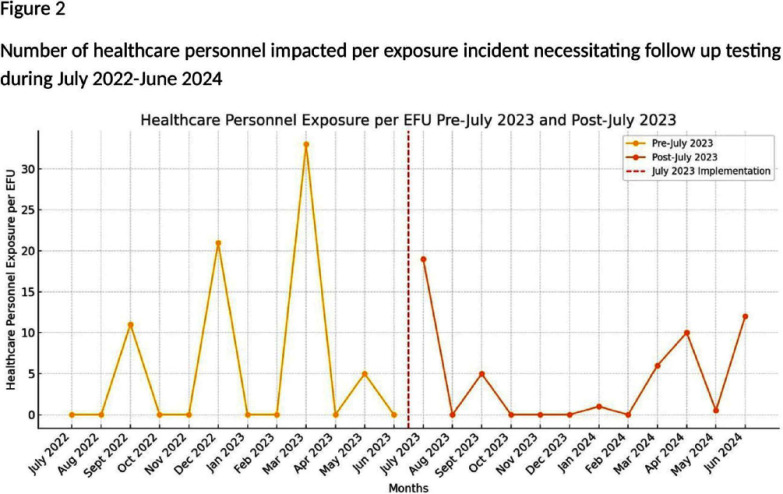

**Figure:**